# Bonding Protocols for Lithium Disilicate Veneers: A Narrative Review and Case Study

**DOI:** 10.3390/biomimetics10030188

**Published:** 2025-03-19

**Authors:** Silvia Rojas-Rueda, Jose Villalobos-Tinoco, Clint Conner, Staley Colvert, Hamid Nurrohman, Carlos A. Jurado

**Affiliations:** 1 Division of Dental Biomaterials, The University of Alabama at Birmingham School of Dentistry, Birmingham, AL 35233, USA; 2 Postgraduate Program in Periodontology and Implant Dentistry, School of Dentistry, National University of Rosario, Rosario S2002KTT, Argentina; 3 Private Practice, Culiacan 80030, Sinaloa, Mexico; 4 Department of General Dentistry, The University of Tennessee Health Science Center College of Dentistry, Memphis, TN 38104, USA; 5 Department of Restorative Dentistry & Prosthodontics, The University of Texas School of Dentistry, Houston, TX 77054, USA; 6 School of Dentistry, Ponce Health Sciences University, Ponce 00716, Puerto Rico

**Keywords:** bonding, ceramics, veneers, esthetic dentistry

## Abstract

Background: The bonding protocol for lithium disilicate veneers in the esthetic zone plays a crucial role in modern dental restoration techniques, focusing on the replication of natural tooth properties and esthetics. This process involves several meticulous steps on both ceramic and tooth surfaces to optimize material performance and bond strength. Methods: The objective of this article is to provide an updated review of the literature on the clinical steps for bonding lithium disilicate veneers in the anterior dentition and to document a clinical case where these advanced restorative techniques were applied to treat a female patient seeking to improve her smile. A preliminary review was conducted on the existing literature regarding the clinical protocols for bonding lithium disilicate veneers in the esthetic zone. The main advantage of careful bonding procedures is that they maximize the full potential of the materials’ properties. Results: A review of the literature reveals some minor differences in cleaning the veneers prior to cementation and in the number of steps involved when combining certain materials in a single application process. However, well-executed bonding procedures, following the manufacturer’s recommendations, can maximize the adhesion between the ceramic and the tooth, allowing the restorations to meet the patient’s esthetic demands. Conclusions: Effective bonding of lithium disilicate veneers in the esthetic zone requires multiple treatments on both the ceramic and tooth surfaces. When procedures are followed carefully, long-term esthetic and functional outcomes can be achieved. It is essential that clinicians are familiar with these steps. Proper patient selection, thoughtful treatment planning, and methodical execution of the case can lead to highly esthetic results that satisfy the patient’s demands and ensure long-term success.

## 1. Introduction

A person’s appearance and esthetic qualities play a significant role in shaping their identity and cannot be overlooked. A radiant smile with bright, white teeth, complemented by well-defined lips, can enhance one’s youthful appearance and increase social attractiveness [1]. High-demand procedures in this domain include the application of veneers to achieve a “Hollywood smile”, orthodontic interventions for dental alignment, and orthognathic surgery for addressing severe dentofacial discrepancies that cannot be corrected through orthodontics alone. These procedures may involve corrective bone surgeries to modify jaw mobility [2,3]. Research has demonstrated a strong link between a beautiful smile and enhanced self-esteem [4]. A recent study examined the impact of facial components on smile attractiveness in 60 individuals. For men, the smile accounted for 49% of attractiveness, with eyes and hair contributing 22% and 6%, respectively. In women, the smile was responsible for 69% of the variation in attractiveness. The study highlighted a strong link between facial and smile attractiveness [5]. When individuals are satisfied with their smile, they tend to have a more positive self-image, which can lead to higher self-worth and a more hopeful perspective on life. Dental veneers are a conservative treatment and have been shown to fulfill patients’ esthetic demands.

Dental veneers, crafted from either resin composite or ceramics, are highly effective in addressing patients’ esthetic desires and enhancing occlusal function [6,7]. While both materials offer excellent results, resin composite is more susceptible to staining, while ceramics provide superior fracture resistance [8,9]. Among the various ceramic options available—such as lithium disilicate, porcelain, leucite, zirconia, and hybrid materials—clinicians must be mindful that bonding protocols differ based on the material chosen [10,11]. Lithium disilicate, in particular, has gained widespread popularity among clinicians and technicians for fabricating laminate veneers due to its ability to meet both functional and esthetic requirements [12,13].

Lithium disilicate veneers have demonstrated excellent long-term results in clinical studies. A recent retrospective clinical evaluation of lithium disilicate restorations found high survival rates over an extended follow-up period [14]. Additionally, a systematic review and meta-analysis on the survival and complication rates of lithium disilicate veneers concluded that this material is highly effective for long-term success [15]. Lithium disilicate veneers can be fabricated using either traditional or CAD/CAM digital workflows. A recent randomized controlled clinical trial comparing both methods showed no statistically significant differences between the two groups after follow-up assessments [16].

The bonding protocols for ceramics can vary depending on the material used, and even within the category of lithium disilicate, these protocols may differ based on the manufacturer’s specific guidelines and recommendations. It is important for clinicians to recognize that bonding is more effective on enamel than on dentin, making minimally invasive tooth preparations essential [17,18]. Several key factors must be carefully considered by clinicians to ensure the successful application of lithium disilicate veneers, including proper tooth preparation with a minimally invasive approach, appropriate treatment of the dental ceramic before cementation, preparation of the tooth surface before cementation, and the selection of the luting cement. The clinical success of laminate veneers is largely determined by selecting the correct indications for the patient, as well as the proper application of materials and techniques that align with the esthetic treatment goals. Given the complexity of bonding protocols, younger clinicians may find them difficult to navigate. This manuscript provides a concise brief review of bonding protocols used in case reports from the literature and includes a case study that outlines the steps and evaluates the clinical workflow required to achieve optimal esthetic results that meet the patient’s functional and esthetic needs.

## 2. Materials and Methods

### 2.1. Review of the Literature

Debonding is a significant complication when bonding ceramic veneers, especially in the esthetic zone, and can result from several factors. Inappropriate case selection, such as poor oral hygiene and gingivitis in the patient, can lead to bleeding during cementation. Improper provisional veneers, with thick margins and rough surfaces, promote food accumulation, contributing to bleeding. Previous debonding may occur if old veneers were removed mechanically, exposing the dentin without the use of laser technology. Poor communication with the dental laboratory, such as an incorrect ratio of veneer thickness to die spacer (which should not exceed 1/3 of the veneer thickness), can also cause debonding or fractures. The use of polishing pastes containing fluoride or oil may interfere with bonding protocols, and improper isolation or tissue management—resulting in sulcular fluids, saliva, or bleeding—can further compromise the bond [19].

A comprehensive search was conducted in December 2024 using MEDLINE (PubMed) and Google to identify relevant articles published between January 1980 and December 2024. The search terms included “lithium disilicate veneers case report” AND (“case report” OR “esthetic veneers”) AND “labial LD veneers case study” (“labial veneer” OR “facial veneer”). Only case reports and case studies detailing the clinical steps involved in the bonding procedure of labial lithium disilicate veneers were considered. Articles included in this review were limited to those written in English. Excluded from the review were letters, book chapters, reviews, and cases for which the full description of the bonding steps was unavailable as shown in Table 1.

Only publications addressing the clinical steps for bonding lithium disilicate veneers were analyzed. Studies related to lithium disilicate crowns or veneers bonded to implant crowns or abutments were excluded. The study selection process was carried out independently by two reviewers (C.A.J. and H.N.), with any disagreements resolved by a third reviewer (S.M). No quantitative analysis, such as meta-analysis, was conducted.

### 2.2. Case Study

**Figure 1:**
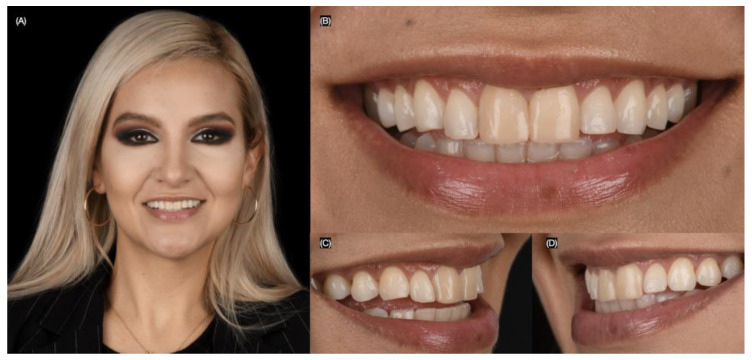
Initial extra-oral situation. (**A**) Face smiling, (**B**) close up, (**C**) right, and (**D**) left side of the smile.

**Figure 2:**
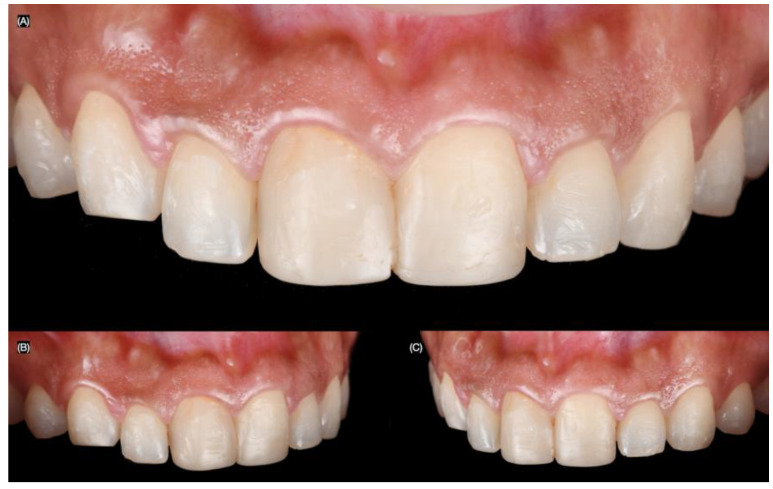
Initial intra-oral situation. (**A**) Initial frontal, (**B**) right, and (**C**) left side intra-oral view.

A 27-year-old female patient presented to the clinic with the chief complaint of wanting to improve her smile. She reported having resin composite restorations placed on her anterior teeth 4 years ago, but she disliked the staining and shape of the restorations. After a clinical evaluation, the patient presented with incisal wear from the right canine to the left canine, stained composite veneers on both central incisors, and spaces between the right canine, right lateral incisor, and right central incisor (Figure 1 and Figure 2).

**Figure 3:**
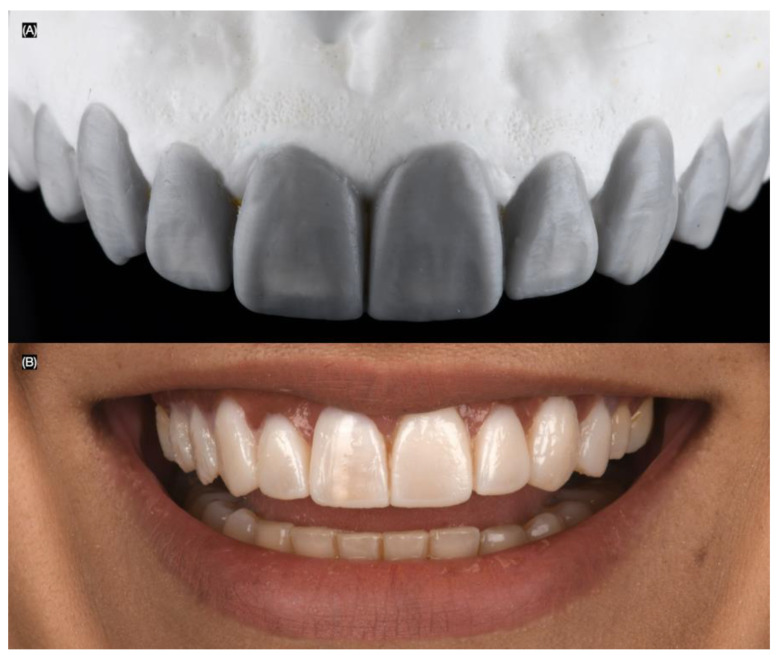
(**A**) Diagnostic wax-up and (**B**) intra-oral mock-up.

The patient was offered tooth whitening to improve the shade and direct resin composite veneers or minimally invasive tooth preparations for ceramic veneers for the anterior teeth. However, the patient declined the bleaching treatment and also rejected resin composite veneers due to previous staining issues, and patient preferred minimally invasive veneer preparations and ceramic laminate lithium disilicate veneers for the anterior dentition. The patient was informed that a diagnostic wax-up would be performed, followed by an intra-oral mock-up, allowing her to physically evaluate the proposed restorations. Based on smile analysis, 10 veneer restorations were deemed necessary. After the diagnostic wax-up (Wax GEO Classic, Renfert, Hilzingen, Germany) and mock-up (Integrity, Dentsply Sirona, Charlotte, NC, USA) were completed, the patient liked the results and approved the plan for lithium disilicate veneers from the right second premolar to the left second premolar (Figure 3).

**Figure 4:**
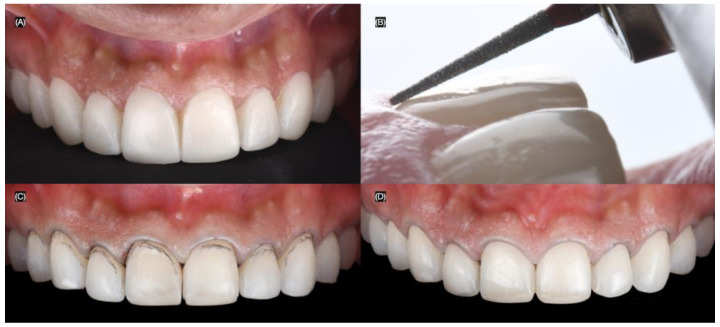
Tooth preparations. (**A**) Intra-oral mock-up prior preparations, (**B**) preparation’s lateral view, (**C**) tooth preparations process, and (**D**) final tooth preparations with #000 cord packed.

At the following appointment, the mock-up was reinserted, and minimally invasive butt joint tooth preparations were performed using a specialized bur kit (Solution Laminate Veneer Preparation System, Brasseler, Savannah, GA, USA) for veneer preparations. A double-zero cord (Ultrapak #00, Ultradent, South Jordan, UT, USA) was packed around all teeth to create the equigingival finish margin for the veneer preparations (Figure 4).

**Figure 5:**
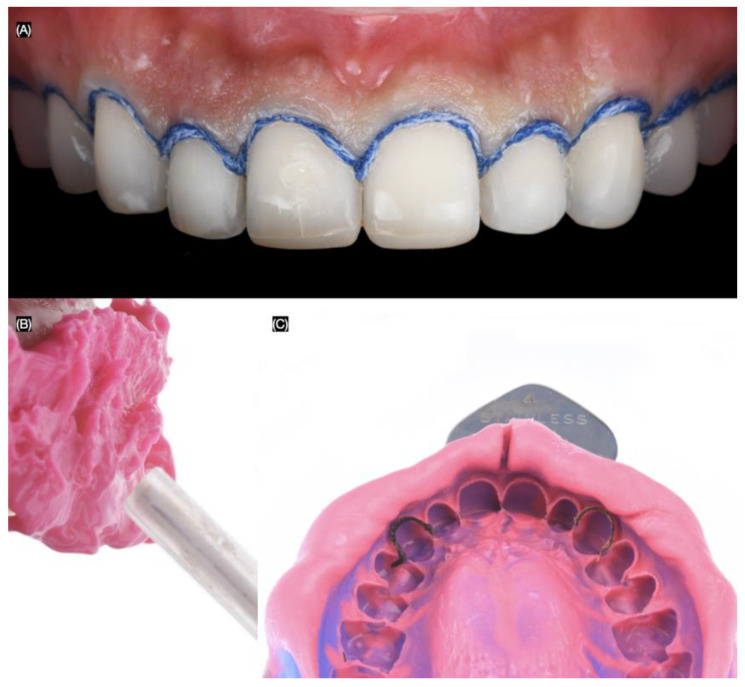
Final impression. (**A**) #00 cord packed frontal view, (**B**) applying light body impression material, and (**C**) final impression.

Final tooth preparations were polished with polishing disks (Sof-Lex XT Disc, 3M, St Paul, MN, USA). A double cord (Ultrapak #0, Ultradent, South Jordan, UT, USA) was used, and a final impression was made with polyvinyl siloxane impression material in both heavy-body and light-body consistency (Figure 5).

**Figure 6:**
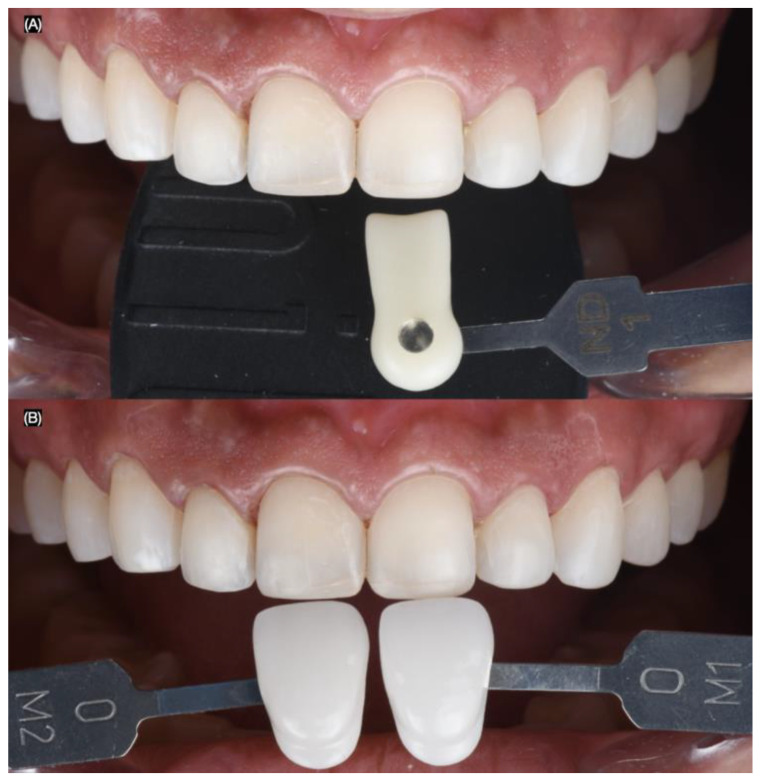
Dental shade photography. Frontal images with (**A**) dentin and (**B**) enamel shade guides.

Intra-oral photographs (Nikon D650) were taken using dentin (IPS Style Ceram Dentin Shade Guide, Ivoclar Group, Schaan, Liechtenstein) and enamel (A–D Shade Guide, Ivoclar Group, Schaan, Liechtenstein) shade guides, both with and without a black background, to evaluate the shade and substrate (Figure 6).

**Figure 7:**
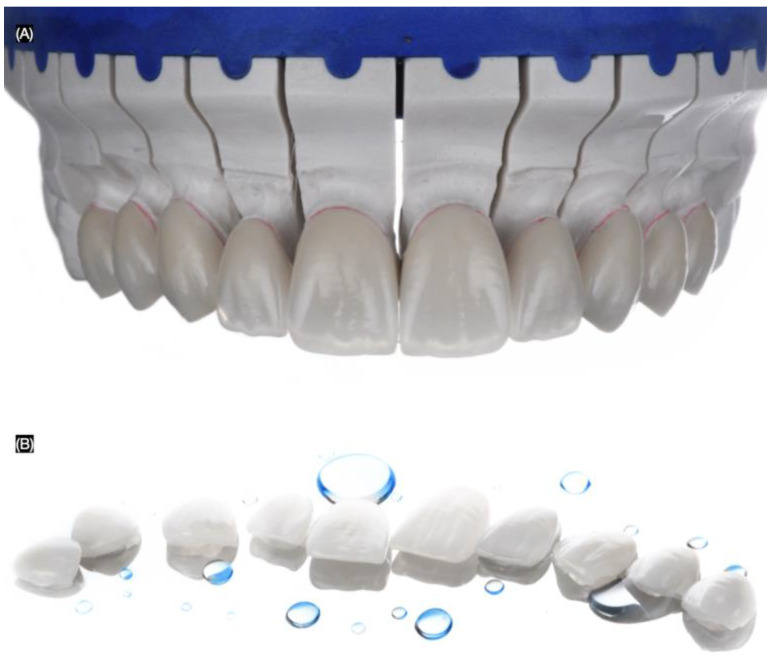
Lithium disilicate veneers. (**A**) Frontal view in master cast and (**B**) in a mirror.

The final master cast was fabricated using type IV stone (Fujirock, GC, Tokyo, Japan), and the ten handcrafted lithium disilicate veneers were fabricated using IPS e.max Ceram (Ivoclar Group, Schaan, Vaduz, Liechtenstein) (Figure 7).

A dry try-in of the ceramic veneers was performed so the clinician and patient could evaluate the margins, shade, and shape of the restorations. Additionally, a try-in with try-in paste in neutral and warm shades (Variolink Esthetic Try-in, Ivoclar Group, Schaan, Vaduz, Liechtenstein) was carried out. The patient and clinician selected a light shade of cement, and the patient requested the procedure to proceed.

First, the ceramic restorations were treated on the intaglio surface with hydrofluoric acid (Porcelain Etch, Ultradent, South Jordan, UT, USA) for 20 s, then rinsed and dried. This was followed by the application of silane (Monobond Plus, Ivoclar Group, Schaan, Vaduz, Liechtenstein) for 60 s. Finally, adhesive (OptiBond FL, Kerr, Orange, CA, USA) was applied, and excess was removed with air. Total isolation was achieved with a dental dam (Dental Dam, Nic Tone, Bucharest, Romania) from the maxillary right first molar to the left first molar, retained with clamps (Clamp #00, Hu-Friedy, Chicago, IL, USA) for optimal isolation.

**Figure 8:**
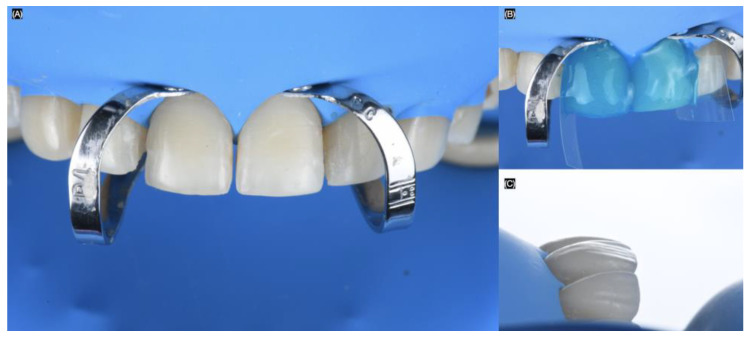
Bonding of the veneers under dental dam isolation. (**A**) Isolation and clamp in central incisors, (**B**) acid etching process, and (**C**) final with dental dam lateral view.

Clamps (Clamp B4, Brinker Hygienic, Coltene, Altstätten, Switzerland) were placed around the gingival contours of each tooth to be treated. The sequence began with both maxillary central incisors. The teeth were sandblasted with water and 29-micron aluminum oxide particles (AquaCare, Velopex, London, UK). Next, the teeth were treated with 37% phosphoric acid (Total Etch, Ivoclar Group, Schaan, Vaduz, Liechtenstein) for 15 s, gently air-dried, and primed with (Optibond FL, Kerr, Orange, CA, USA). Excess was removed, and finally, a light-shade luting resin cement (Variolink Esthetic LC, Ivoclar Group, Schaan, Vaduz, Liechtenstein) was applied to bond the lithium disilicate veneers. The excess cement was removed, and each restoration was cured for 20 s on the facial, 20 s interproximal, 20 s mesial, and 20 s distal (Figure 8).

**Figure 9:**
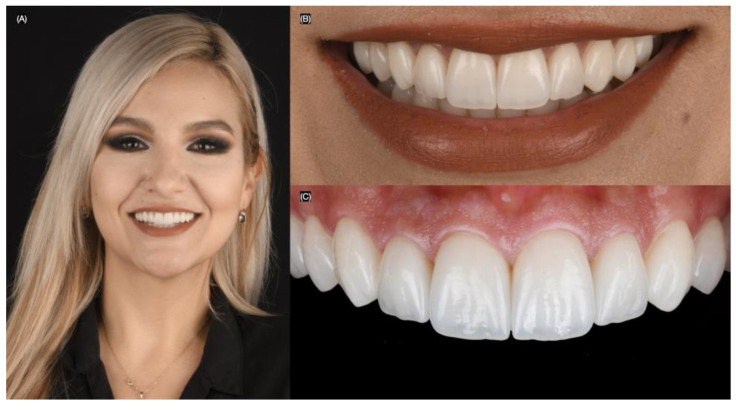
Final bonded restorations. (**A**) Face smiling, (**B**) close-up of the smile, and (**C**) intra-oral.

**Figure 10:**
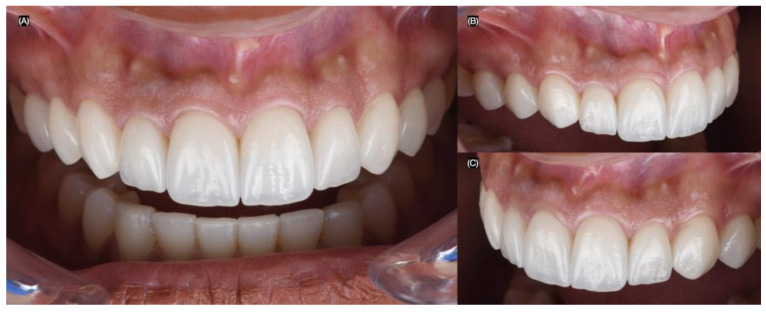
Final intra-oral. (**A**) Frontal, (**B**) right, and (**C**) left side view.

The patient was pleased with the contours, shape, and shade of the final lithium disilicate veneers. She was provided with an occlusal night guard to protect the restorations at night (Figure 9 and Figure 10).

**Figure 11:**
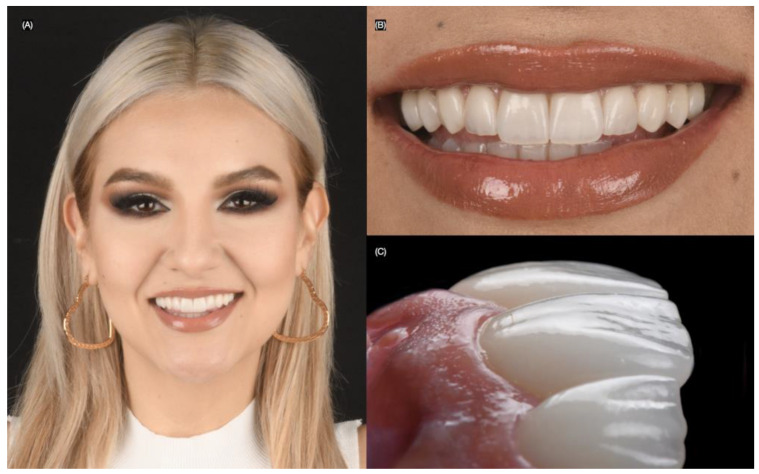
Two years follow-up. (**A**) Face smiling, (**B**) close-up of the smile, and (**C**) close-up lateral view.

At the two-year follow-up, the patient remained satisfied with the clinical outcome (Figure 11).

## 3. Results

The literature offers several case reports on lithium disilicate veneers in the esthetic zone; however, very few meticulously describe each bonding step during the cementation procedure. The findings from our brief literature review of articles detailing all the clinical steps for placing lithium disilicate ceramic veneers are presented in Table 2.

The findings from the literature review of systematic reviews and retrospective clinical studies evaluating the survival rate of lithium disilicate veneers are presented in Table 3.

**Figure 12:**
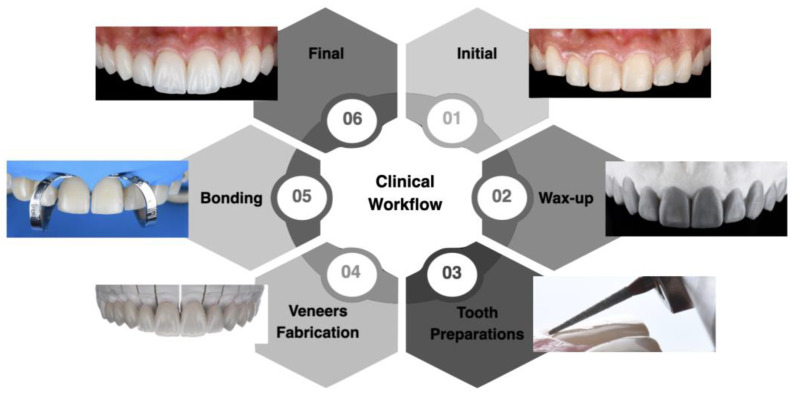
Summary of the clinical study workflow.

The clinical workflow implemented allowed for proper planning and execution of minimally invasive lithium disilicate veneers in the esthetic zone. The diagnostic wax-up and mock-up provided the patient with an opportunity to assess the desired outcome before any invasive procedure. The bonding procedure, performed under total isolation with a dental dam, prevented contamination and maximized the effectiveness of the materials used. The patient was satisfied with the results, and at the two-year follow-up appointment, both the restorations and surrounding tissue continued to meet the patient’s esthetic and functional needs. A flowchart outlining the steps of the clinical workflow can be seen in Figure 12.

## 4. Discussion

### 4.1. Tooth Preparation for Labial Veneers

The preparation of the teeth plays a significant role in the durability and appearance (including translucency and color) of the ceramic restoration. The way the tooth is prepared affects both the internal contour and the thickness of the ceramic material. If the tooth presents staining, it is recommended to undergo whitening before placing the ceramic veneer to achieve optimal esthetic results [32,33,34,35]. In cases where tooth whitening is not feasible due to time limitations or patient preferences, the restoration should be made thicker to properly match the underlying tooth color. A recent in vitro study evaluated how material thickness and opacity impact the restoration of discolored teeth. Ninety lithium disilicate veneers and sixty resin composite veneers were fabricated at 0.7, 1.0, and 1.2 mm thicknesses and cemented onto 150 human premolars. The results showed that 1.2 mm veneers effectively masked discolored substrates, influencing the final restoration color [36].

Another crucial factor in tooth preparation is minimizing invasiveness, as it has been shown that bonding to enamel provides greater bond strength than bonding to dentin [36,37]. Therefore, the use of tooth reduction guides is strongly recommended to help clinicians control the removal of tooth structure [37,38]. Studies have demonstrated that tooth reduction guides offer more precise control and require less tissue removal compared to traditional free-hand preparations [39,40].

### 4.2. Lithium Disilicate Ceramic Veneers

Lithium disilicate has gained significant popularity, particularly in highly esthetic cases, due to its excellent durability and versatility across various clinical applications. As clinicians, technicians, and ceramists, it is crucial to be well-versed in using this material by understanding the intricate details of its construction [40,41,42]. Numerous clinical studies have demonstrated impressive long-term survival rates for lithium disilicate veneers [15,28,29,30,31]. A recent clinical study assessed 79 multilayered lithium disilicate veneers placed on anterior teeth, with an average follow-up of 3 years. The results revealed a 98.7% survival rate, with only one instance of restoration detachment during the 3-year period [29]. Additionally, a retrospective study analyzed the performance of 197 CAD/CAM lithium disilicate veneers placed in 32 patients, with a 10-year follow-up. The study reported a 92.7% survival rate, confirming that lithium disilicate has excellent long-term durability [31]. Lastly, a systematic review and meta-analysis, which included over 29 studies, evaluated the survival rate of lithium disilicate laminate veneers. The analysis concluded a 96.81% survival rate over an average period of 10.4 years, reinforcing the material’s strong long-term performance [15].

### 4.3. Ceramic Surface Treatment

Proper etching of the ceramic surface is a critical step for the clinical success of both indirect ceramic bonded restorations and direct ceramic repair procedures. Etching alters the surface topography, leading to an increase in surface area and changes in the wetting behavior of the porcelain [43]. The method of treating the ceramic surface varies depending on its composition, with recommended times outlined in Table 4 [44].

Hydrofluoric acid conditioning is effective in eliminating surface imperfections and smoothing out any remaining defect tips, which reduces stress concentration and enhances the overall strength of the material. This process is similar across different ceramics, such as feldspathic, leucite, and lithium disilicate-reinforced ceramics [44,45,46,47]. Silanization of the etched porcelain with a bifunctional coupling agent creates a chemical bond between the porcelain and the resin composite. One end of the silane group bonds chemically with the hydrolyzed silicon dioxide on the ceramic surface, while the methacrylate group on the other end copolymerizes with the adhesive resin [48]. Single-component systems contain silane dissolved in alcohol or acetone and require prior acidification of the ceramic surface with hydrofluoric acid to activate the chemical reaction. In contrast, two-component silane systems mix the silane with an aqueous acid solution to hydrolyze the silane, enabling it to directly bond with the ceramic surface [49].

### 4.4. Tooth Surface Treatment

The enamel surface should be treated with 37% phosphoric acid, which increases its surface energy and promotes optimal bonding by ensuring the adhesive wets the surface effectively [50]. During this process, it is essential to prevent contamination from saliva or moisture from the breath, as this can decrease the enamel’s surface energy and compromise the bonding. Phosphoric acid etching creates a “frosty” appearance on the enamel surface, which indicates a successful procedure due to the enamel’s inorganic composition and its ability to etch well. In contrast, bonding to dentin is more challenging to control because of its complex structure, which includes both inorganic and organic components as well as tubules [51]. The variety of dentin-bonding agents available and the different bonding techniques can cause confusion in clinical practice, so it is highly advised to follow the manufacturer’s guidelines to ensure success. Furthermore, dentin-bonding systems are very sensitive to technique, and achieving reliable results becomes more difficult when optimal moisture control cannot be maintained [52].

### 4.5. Tooth Isolation

It is well-established that clean, unblemished enamel offers the most reliable surface for bonding ceramic veneers, making conservative tooth preparation and complete isolation using a rubber dam essential for ensuring the long-term success of the restoration, as demonstrated by five years of follow-up. Rubber dam isolation offers numerous benefits for the clinician, including preventing contamination from saliva, blood, and sulcular fluids, as well as enhancing visibility in the treatment area [53]. Research supports the use of dental dams, with a 2016 Cochrane Library review indicating that, at least for direct restorations, rubber dam use is associated with a reduced failure rate [54]. Additionally, patients have reported greater comfort during dental procedures when a rubber dam is used, compared to when it is not [55]. However, clinicians should be mindful of the materials used in rubber dams, as some patients may experience allergic reactions, particularly to latex. Common symptoms of a latex allergy include skin irritation, redness, rashes, or hives, as well as itching in the nose, throat, or eyes, nausea, abdominal discomfort, and difficulty breathing. Although most reactions occur shortly after exposure, some skin reactions can take up to 24–48 h to manifest [56]. To minimize risk, it is advisable to use non-latex dental dams. Ultimately, adhering to conservative protocols, such as minimal enamel preparation and complete isolation with a rubber dam before bonding ceramic restorations, enhances the effectiveness of the bonding system, as demonstrated in cases involving ceramic veneers.

### 4.6. Luting Cements

The clinical success of laminate veneers largely depends on the cementation process of the indirect restorations, along with other factors [57]. Given the brittle nature of ceramics, adhesive cementation is employed to enhance fracture resistance by infiltrating internal surface imperfections, reducing crack propagation, and facilitating better stress transfer from the restoration to the tooth structure [58]. Luting cements are versatile materials that can achieve excellent esthetic outcomes. Resin cements, known for their strong retention and fracture resistance, are often used, although the adhesive cementation technique is sensitive and can result in a higher incidence of postoperative sensitivity [59]. Luting cements can be divided into two categories: (1) cements that require the use of conventional or self-etching adhesives, and (2) self-adhesive cements that do not require prior conditioning of the tooth structure [60]. Ideal cement properties include the ability to form a stable bond between the restorative material and the tooth surface; resistance to both tensile and compressive forces; appropriate elasticity; optimal viscosity for the cementation line thickness and proper restoration seating; and biocompatibility [61]. These characteristics are crucial for the longevity of the restoration, as they effectively prevent microleakage, fracture, or displacement of the restoration. For the cementation of porcelain veneers, light-curing luting composites are typically preferred. A key advantage of light-curing is the extended working time it provides compared to dual-cure or chemically cured materials. This allows the dentist to remove excess composite before curing, significantly reducing the finishing time required for these restorations. Additionally, light-cured composites offer superior color stability when compared to dual-cure or chemically cured systems [62].

### 4.7. Case Report

This case report aims to demonstrate the clinical procedures involved in addressing esthetic concerns, including the diagnostic evaluation, diagnostic wax-up, intra-oral mock-up, minimally invasive tooth preparation on the mock-up, shade selection using photographs, fabrication of hand-crafted lithium disilicate veneers, and cementation of the veneers under complete isolation with a dental dam. The patient in this case presented with aged and stained resin composite veneers on both central incisors, along with mild incisal wear extending from the right canine to the left canine. She sought to enhance her smile, and because of her wide smile that displayed up to the second premolars on both sides, a treatment plan involving minimally invasive veneer restorations for all the visible teeth was proposed and accepted. The diagnostic wax-up, followed by the mock-up, gave the patient a chance to physically evaluate the potential restorations, which she approved, and she requested to proceed with treatment. Tooth preparations were carried out directly on the mock-up to ensure precise tooth reduction while preserving enamel. The custom-made hand-crafted veneers delivered excellent esthetic results due to their ability to closely mimic the natural microstructure of teeth. The bonding process was conducted under total isolation with a dental dam to prevent contamination and optimize the adhesive bond and resin cement properties. At a two-year follow-up, the patient remained satisfied with the results.

The review protocol in the case study involved first treating the ceramic veneers with hydrofluoric acid for 20 s, followed by the application of silane for 60 s. Next, an adhesive was applied and then air-dried. For the tooth treatment, the surface was sandblasted with water and aluminum oxide particles for 15 s, followed by the application of a primer for 15 s, and finally, the veneer was cemented with resin cement. The application times for each material in the case study were based on the manufacturers’ recommendations. As noted in the literature review of clinical cases, there is some variation in application times, which can be attributed to differences in manufacturers’ guidelines and whether clinicians use primer and adhesive separately or in a single-bottle application.

### 4.8. Limitations

This brief narrative review has some limitations, such as relying on only one database and using a focused search strategy, which does not align with the Preferred Reporting Items for Systematic Reviews and Meta-Analyses (PRISMA) guidelines. The limitations of a single case report include the lack of quantitative analysis, with the qualitative analysis being based on the patient’s self-reported satisfaction with the results. Additionally, the report only includes a 2-year follow-up, so future case studies should provide longer follow-up periods. Finally, future research should also focus on comparing different adhesive brands for placing lithium disilicate veneers in patients.

## 5. Conclusions

Dental care using lithium disilicate veneers in the esthetic zone is an effective, minimally invasive approach to restore teeth with mild wear, gaps between teeth, and to improve the shade and contours of the teeth. Clinicians must be aware that the bonding protocol involves both treating the tooth and the ceramic before the final step of cementing the restoration. This process is recommended to be executed under dental dam isolation. It is essential for clinicians to carefully read the manufacturer’s recommendations in order to maximize the properties of the materials.

## Figures and Tables

**Table 1:** Inclusion and exclusion criteria of the clinical cases search.

Inclusion	Exclusion
Clinical case reports	In vitro studies
Labial lithium disilicate veneers	Retrospective studies
Describing all the bonding steps	Reviews
	Veneers on implant abutments/crowns
	Cases without description of the bonding steps

**Table 2:** Publications on clinical bonding protocols for lithium disilicate veneers in the esthetic zone [20,21,22,23,24,25,26,27].

Authors, Year, and Title of Publication	Type of Restorations	Bonding Protocol	Results
Schmitter M et al., 2012. Minimally invasive lithium disilicate ceramic veneers fabricated using chairside CAD/CAM [20].	A thin (0.4 mm) lithium disilicate veneer to correct the shape of right lateral incisor.	– The veneer received 35% phosphoric acid (UltraEtch) for 20 s, then silanized for 1 min (Monobond S). – Teeth were etched with 37% phosphoric acid (Total Etch) for 60 s in non-prepared areas and 30 s in prepared areas. Primer and adhesive (Syntac) were applied and restorations cemented with resin cement (Variolink).	After one year, the patient was recalled. The restoration was intact without any chipping, discoloration, or other complications.
Soares PV et al., 2014. Esthetic rehabilitation with laminated ceramic veneers reinforced by lithium disilicate. [21].	Four lithium disilicate veneers to close spaces between mandibular central and lateral incisors.	– Veneers were treated with 9.5% hydrofluoric acid (Porcelain Etch) for 20 s, and then cleaned with 37% phosphoric acid (Total Etch) for 60 s. Restorations were silanized (Monobond Plus) for 60 s. – Teeth received 37% phosphoric acid (Total Etch) for 30 s. Adhesive agent (ExciTE F DSC) was applied and photo-cured for 20 s. A photo-cure resin cement (Variolink Veneer) was used to cement the restorations.	Detailed planning, correct selection of dental materials, and quality communication with the prosthetic technician contributed to a harmonious smile and the evident satisfaction of both patient and professionals.
Zandinejad A et al., 2015. Digital workflow for virtually designing and milling ceramic lithium disilicate veneers [22].	Six lithium CAD/CAM lithium disilicate veneers from canine to canine.	– The veneers were etched with 10% hydrofluoric acid for 25 s and silanized (Ceramic Silane). – They were then cemented with light-cure resin cement (RelyX Veneer).	All restorations fabricated were clinically acceptable in terms of marginal fit, shape, contour, and esthetics.
Martins JD et al., 2019. Digital smile designing, pressing and stratifying ceramic lithium disilicate veneers to rehabilitate dental agenesis [23].	Eight lithium disilicate veneers from right first premolar to left first premolar on a patient to replaced stained resin composites.	– Restorations were treated with 5% hydrofluoric acid, the silanized (RelyX Ceramic Primer), subjected to silane heat treatment with a hair dryer in the hot air function and then submerged in a 4 min ultrasonic bath. – The restorations were cemented with a light-cured cement (RelyX Veneer) and adhesive (Single Bond Universal).	Ceramic veneers are a possibility for conservative and successful treatment of smile anatomization in patients with dental agenesis using careful treatment planning.
Souza R et al., 2020. Two-year follow-up of ceramic veneers and a full crown treated with self-etching ceramic primer [24].	Five ultra-thin lithium disilicate veneers and one full crown in the esthetic zone.	– The restorations were treated with Monobond Etch and Prime (MEP) for 20 s using a microbrush, allowing the product to act for an additional 40 s. Then, the they were rinsed and dried. – The teeth were then treated with 35% phosphoric acid for 20 s. Finally, an adhesive (Excite F) was applied to the prepared teeth, and the restorations were cemented with resin cement (Variolink).	The satisfactory clinical performance of lithium disilicate restorations treated with Monobond Etch and Prime (MEP) after two years of follow-up supports the promising results shown in laboratory studies.
Tyagi R et al., 2022. Lithium disilicate (“IPS” e. Max computer-aided design) veneers for the esthetic rehabilitation in a young adolescent [25].	Four lithium disilicate veneers on central and lateral incisors on a patient with generalized Fluorosis Grade 3 and Class I fracture on right incisor.	– Veneers were treated with 9.5% hydrofluoric acid (Porcelain Etch) for 1 min, followed by a silane coupling agent (Silane Ultradent) for 60 s. – Teeth were treated with 37% phosphoric acid (Total Etch) for 15 s, and then bonding agent (Tetric N-Bond) and polymerized for 40 s. Dual-cure resin cement (RelyX U200) was used for luting the veneers.	Correction of the discoloration by a less invasive technique was achieved. Lithium disilicate (IPS” e. Max) veneers present a promising approach to the management of esthetic concerns in the case of a young permanent tooth.
Das S et al., 2023. A Cosmetic Chronicle: A case report on the treatment of Tetracycline induced discoloration with Lithium Disilicate Veneers [26].	Six lithium disilicate veneers from right canine to left canine on a patient tetracycline discoloration.	– Veneers were treated with 5% hydrofluoric acid (Ultradent) for 20 s. Then, silane coupling agent (Calibra Silane) was applied for 1 min. Adhesive (Prime and Bond NT) was applied and light cured. – Teeth were treated with 37% phosphoric acid for 30 s. Areas where dentin was exposed, the surfaces were treated with 2% chlorhexidine digluconate (CHX) for 20 s. Resin cement (Calibra Cement) was used to cement the restorations.	Restoration of such drug-induced intrinsic discolorations with laminate veneers is a viable and predictable treatment option presently due to intensified cognizance and advancements in the field of esthetic dentistry.
Hu E et al., 2024. Restoring Severe Tetracycline Stained Teeth with Milled Lithium Disilicate Ceramic Veneers [27].	Lithium disilicate veneers from right second premolar to left second premolar on a patient with tetracycline-stained teeth.	– Veneers were treated with 5% hydrofluoric acid (IPS Ceramic Etching Gel) for 20 s then cleaned and silanated (Clearfill Ceramic Primer). – Teeth were treated with 38% phosphoric acid (Etch-Rite) for 15 s. Self-etching adhesive (Panavia V5) applied on teeth for 20 s and veneers cemented with resin cement (Panavia Veneer LC).	This case report demonstrated that monolithic lithium disilicate veneers can completely cover underlying discoloration in a patient with severe tetracycline staining while achieving predictable, esthetic, and life-changing results.

**Table 3:** Reviews and clinical studies evaluating the long-term survival of lithium disilicate veneers [15,28,29,30,31].

Authors, Year and Title of Publication	Methodology	Conclusions
Klein P et al., 2021. Survival and Complication Rates of Feldspathic, Leucite-Reinforced, Lithium Disilicate and Zirconia Ceramic Laminate Veneers: A Systematic Review and Meta-Analysis [15]	Survival and complication rates of leucite-reinforced, lithium disilicate and zirconia veneers. Results: 96.8% survival rate at 10.4 years.	Ceramic laminate-veneers are a reliable treatment option. Lithium disilicate veneers may be preferred as a restorative material for long-term success.
Malchiodi L et al., 2019. Clinical and Esthetical Evaluation of 79 Lithium Disilicate Multilayered Anterior Veneers with a Medium Follow-Up of 3 Years [28]	A total of 79 lithium disilicate multilayered anterior veneers evaluated with a medium follow-up of 3 years. Results: 98.7% survival rate.	Lithium disilicate veneers in the esthetical rehabilitation of worn anterior teeth proved to be an effective way of treatment in a medium follow-up of 3 years.
Imburgia M et al., 2021. A Retrospective Clinical Study on 1075 Lithium Disilicate CAD/CAM Veneers with Feather-Edge Margins Cemented on 105 Patients [29]	A retrospective clinical study on 1075 lithium disilicate veneers cemented on 105 patients. Results: 99.83% survival rate in observation period of 30.8 months.	Lithium disilicate veneers showed good clinical performance in terms of survival, color matching, ceramic surface, marginal discoloration, and integrity.
Nejatidanesh F et al., 2018. Five year clinical outcomes and survival of chairside CAD/CAM ceramic laminate veneers—a retrospective study [30]	Five-year clinical outcomes and survival of chairside CAD/CAM lithium disilicate veneers—a retrospective study. A total of 197 veneers placed in 71 patients. Results: 97.8% survival rate after 5 years.	Chairside CAD/CAM lithium disilicate offer high survival rate.
Sen N et al., 2024. Retrospective Evaluation of Factors Affecting Long-Term Clinical Performance of CAD/CAM Laminate Veneers [31]	Retrospective evaluation of factors affecting long-term clinical performance of CAD/CAM laminate veneers. A total of 197 laminate veneers placed by a single operator and evaluated for up to 10 years. Results: 89.1% survival rate of E.max CAD lithium disilicate.	CAD/CAM laminate veneers milled from lithium disilicate ceramics have high survival rates.

**Table 4:** Surface treatment protocols according to the type of the ceramic.

Ceramic	Treatment
Feldspathic Porcelain	9.5% hydrofluoric acid for 2 to 2.5 min
Leucite	9.5% hydrofluoric acid for 60 s
Lithium Disilicate	9.5% hydrofluoric acid for 20 s

## Data Availability

Data presented in this study are available on request from the corresponding authors.
